# Supplementary material for: Vision shapes neural maps of space through an ancient midbrain pathway
Source: bioRxiv. 2026 May 18:2026.05.16.725555. Preprint. [Version 1] doi: 10.64898/2026.05.16.725555 (PMC13228374; doi:10.64898/2026.05.16.725555)
Supplement: Supplement 1 [file NIHPP2026.05.16.725555v1-supplement-1.pdf]

bioRxiv preprint doi: https://doi.org/10.64898/2026.05.16.725555; this version posted May 19, 2026. The copyright holder for this preprint (which was not certified by peer review) is the author/funder, who has granted bioRxiv a license to display the preprint in perpetuity. It is made available under a CC-BY 4.0 International license.

Fig. S1

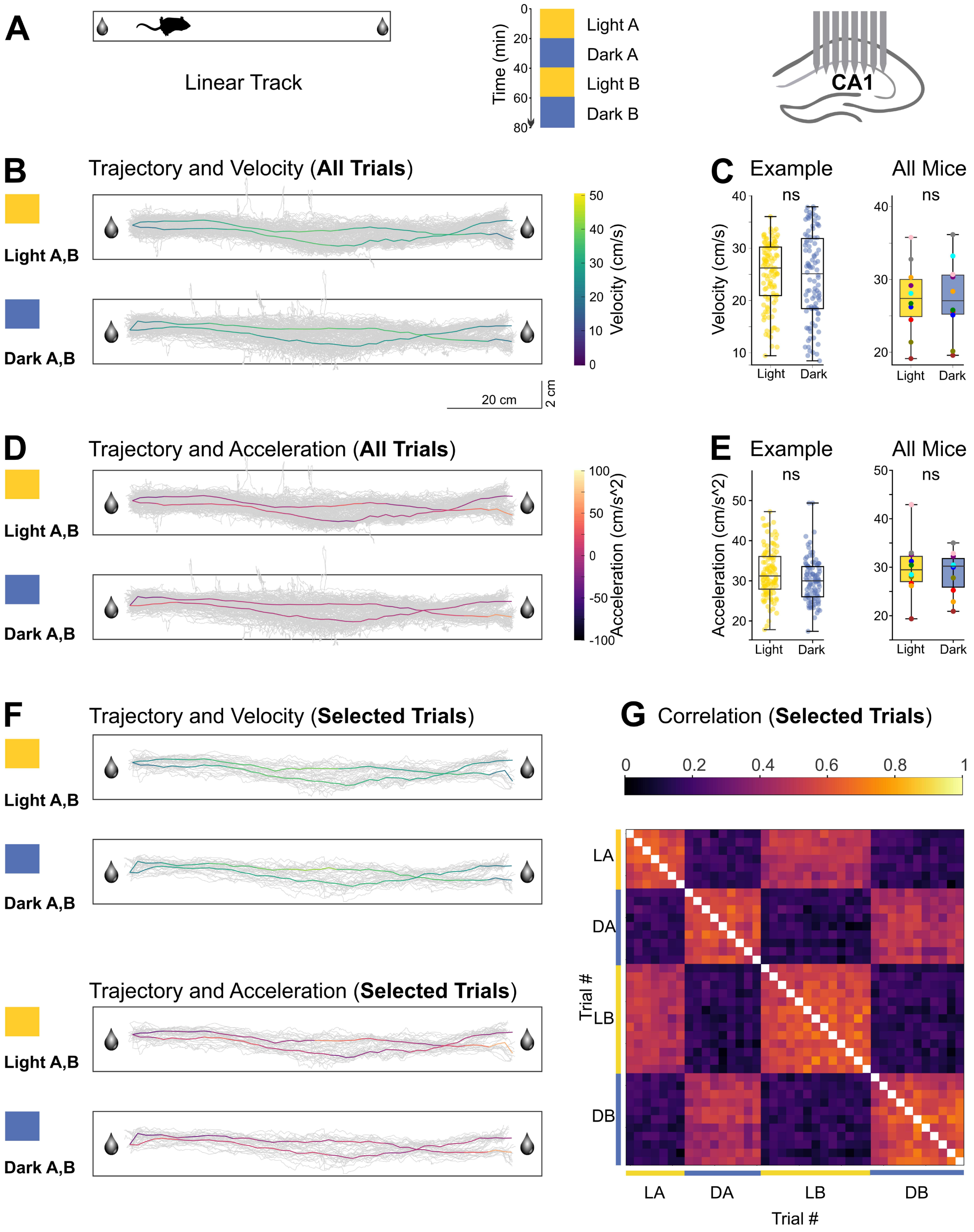

bioRxiv preprint doi: https://doi.org/10.64898/2026.05.16.725555; this version posted May 19, 2026. The copyright holder for this preprint (which was not certified by peer review) is the author/funder, who has granted bioRxiv a license to display the preprint in perpetuity. It is made available under a CC-BY 4.0 International license.

Fig. S1. Light-Dark remapping does not depend on behavioral variability
(A) Schematic of the linear track task (left), experimental timeline showing interleaved light and dark epochs (center), and the recording site in hippocampal CA1 (right). **(B)** Spatial trajectories for Light and Dark conditions. Individual runs are shown in gray. The median trajectory is overlaid and color-coded by velocity. **(C)** Median velocity in Light (yellow) and Dark (blue) epochs for a representative mouse (left; dots represent individual trials) and the population (right; dots represent individual mice). Box plots indicate median and interquartile range. *ns*, not significant (Wilcoxon rank-sum test for single mouse; Wilcoxon signed-rank test for population). **(D)** Spatial trajectories formatted as in (B), but color-coded by acceleration. **(E)** Mean acceleration in Light and Dark epochs, formatted as in (C). **(F)** Trajectories of trials selected for kinematic similarity (see Methods), color-coded by velocity (top pair) and acceleration (bottom pair). **(G)** Pairwise Spearman correlation matrix of spatial population activity from the kinematically matched trials. Trials are arranged chronologically. Labels indicate epochs: LA (Light A), DA (Dark A), LB (Light B), and DB (Dark B).

bioRxiv preprint doi: https://doi.org/10.64898/2026.05.16.725555; this version posted May 19, 2026. The copyright holder for this preprint (which was not certified by peer review) is the author/funder, who has granted bioRxiv a license to display the preprint in perpetuity. It is made available under a CC-BY 4.0 International license.

Fig. S2

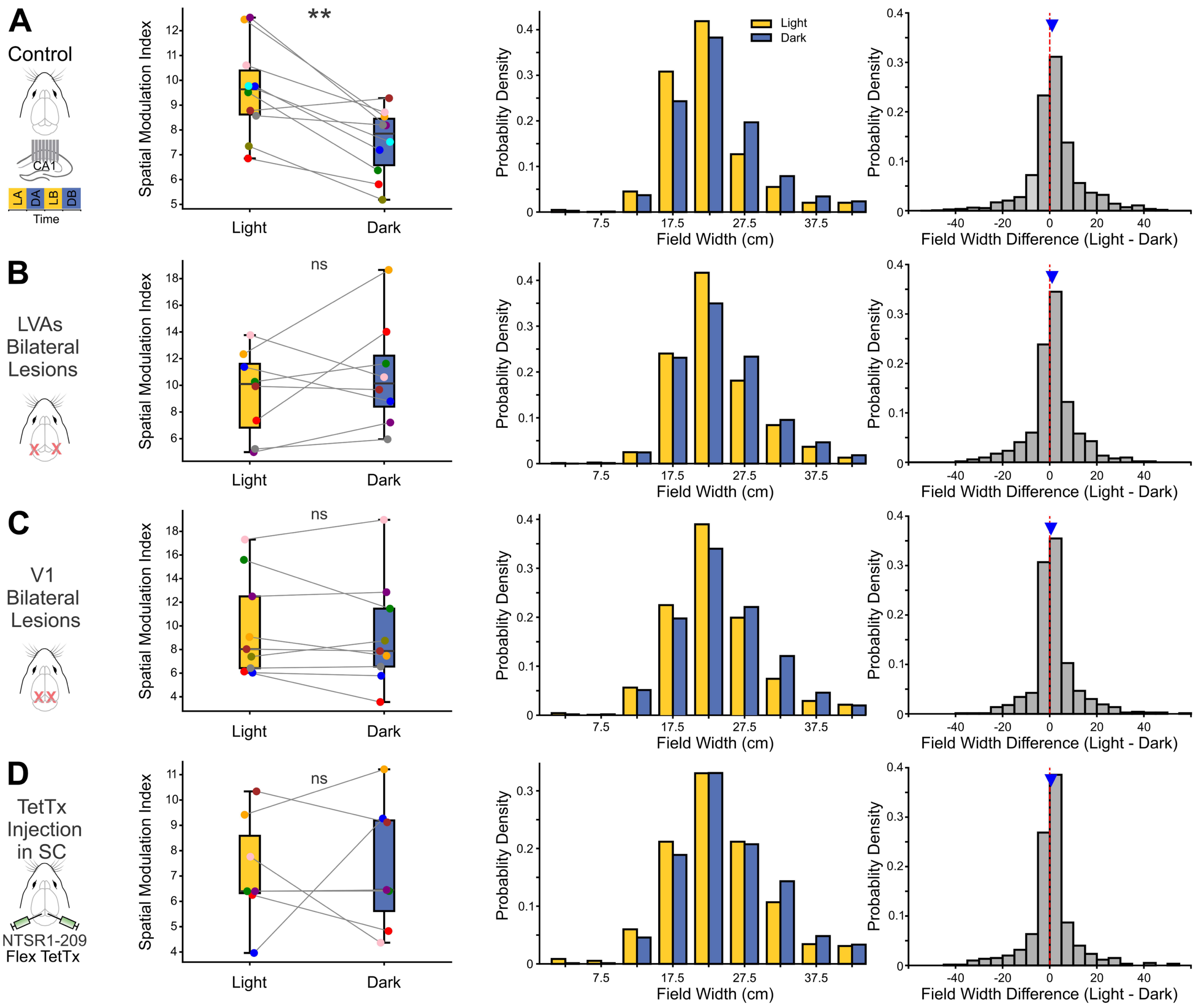

Fig. S2. Stability of place field properties across visual pathway manipulations.
(A) Control mice. Left: Experimental paradigm and median Spatial Modulation Index (SMI) in Light and Dark epochs. Paired lines connect data from individual mice; box plots indicate median and interquartile range. p = 0.006 (Wilcoxon signed-rank test), N = 10 mice. Center: Distribution of place field widths (pooled across mice) in Light (yellow) and Dark (blue) conditions. Right: Distribution of the difference in place cell firing rate (Light − Dark). The blue triangle indicates the population median; the vertical line at 0 indicates no difference. **(B)** Same as (A), for mice with lateral visual cortex lesions. ns, not significant; p = 0.461 (Wilcoxon signed-rank test), N = 8 mice. **(C)** Same as (A), for mice with primary visual cortex (V1) lesions. p = 0.570 (Wilcoxon signed-rank test), N = 9 mice. **(D)** Same as (A), for mice with SC pathway silencing. p = 0.938 (Wilcoxon signed-rank test), N = 7 mice.

bioRxiv preprint doi: https://doi.org/10.64898/2026.05.16.725555; this version posted May 19, 2026. The copyright holder for this preprint (which was not certified by peer review) is the author/funder, who has granted bioRxiv a license to display the preprint in perpetuity. It is made available under a CC-BY 4.0 International license.

Fig. S3

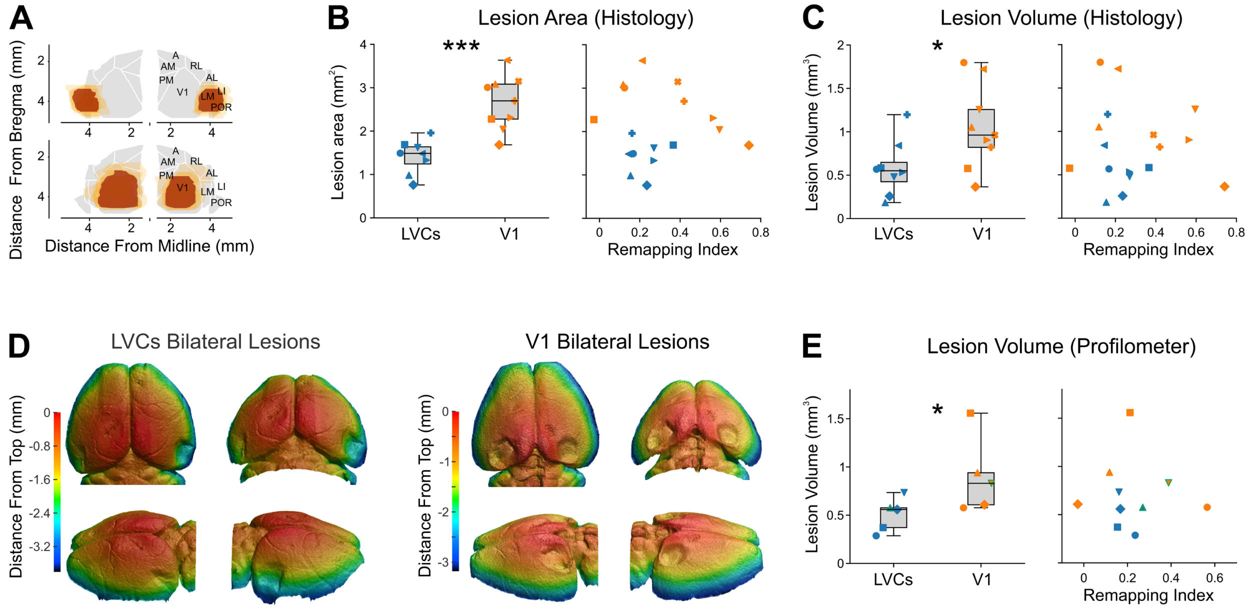

Fig. S3. Histological and laser-profilometry analysis of LVCs and V1 lesions.
(A) Reconstructions from histological data of bilateral LVCs (top, 8 mice) and V1 lesions (bottom, 9 mice) superimposed on a reference map of the visual cortex generated from the Allen Common Coordinate Framework. Dark red indicates the median lesion extent across all mice, overlaid on individual lesion extents (lighter orange). **(B)** Left: Comparison of the areas of LVC and V1 lesions, quantified from histological sections. Box plots indicate median (center line), interquartile range (box edges), and range (whiskers). N = 8 LVC mice, 9 V1 mice; ***, p = 3.29 e-04 (Wilcoxon rank-sum test). Right: Scatter plot correlating V1 and LVC lesion areas with Remapping Index. R = -0.023; p = 0.929 (Pearson's correlation test); ns, not significant. **(C)** Same as in (B) but for the volumes of LVC and V1 lesions measured from histological sections. Left: *, p = 0.036 (Wilcoxon rank-sum test). Right: N = 8 LVC mice, 9 V1 mice; R = -0.092; p = 0.724 (Pearson's correlation test). **(D)** Top, posterior, and side views of LVCs (left) and V1 (right) lesions in two brains digitally reconstructed from laser-profilometry data. **(E)** Left: Same as in (C) but for the volumes of LVCs and V1 lesions measured from laser-profilometry reconstructions. N = 5 LVC mice, 5 V1 mice; *, p = 0.028 (Wilcoxon rank-sum test). Right: Scatter plot correlating V1 and LVC lesions areas with the Remapping Index. Symbols highlighted in green correspond to the examples in (D). N = 5 LVC mice, 5 V1 mice; R = −0.165; p = 0.790 (Pearson's correlation test).

bioRxiv preprint doi: https://doi.org/10.64898/2026.05.16.725555; this version posted May 19, 2026. The copyright holder for this preprint (which was not certified by peer review) is the author/funder, who has granted bioRxiv a license to display the preprint in perpetuity. It is made available under a CC-BY 4.0 International license.

Fig. S4

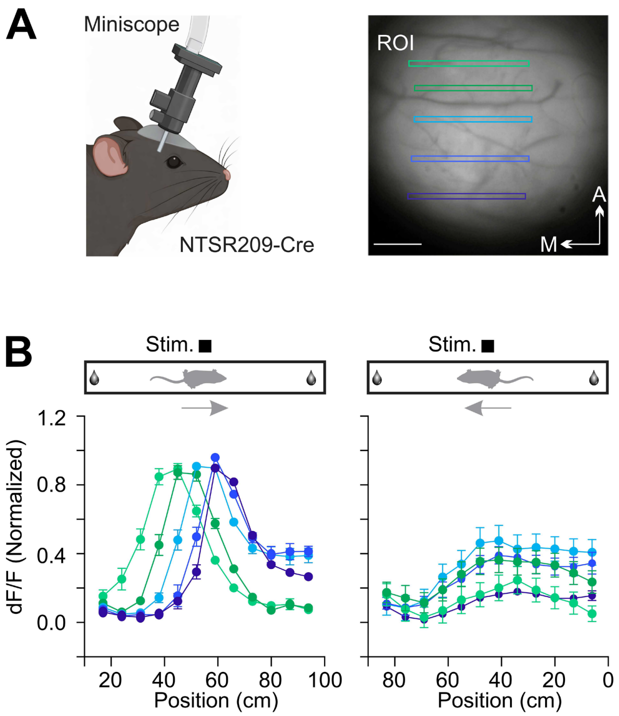

Fig. S4. Hemifield specificity of SC widefield neurons during locomotion
(A) Left: Schematic of the experimental setup showing a Miniscope attached to the head of an NTSR1-GN209-Cre mouse. Right: Representative field of view with color-coded Regions of Interest (ROIs) drawn along the anterior-posterior axis of the SC. Scale bar: 200µm.
(B) Top: Schematics of the linear track showing the position of a static visual stimulus (black square) while the mouse traverses the track in opposite directions. Bottom Left: Normalized fluorescence changes (dF/F) for color-coded ROIs plotted against the animal's position on the track in the forward direction. Bottom Right: Normalized dF/F for the same ROIs plotted against position during traversal in the reverse direction. Error bars indicate SEM.

Movie S1. Calcium imaging of the neuropil of widefield neurons in the superior colliculus during locomotion.
Representative Miniscope imaging of the neuropil of the right superior colliculus (SC) of a Ntsr1–GN209–Cre mouse in which widefield neurons conditionally express GCaMP8m. The animal is traversing a linear track with a static visual cue (black square) positioned in the left hemifield. Note the spatial shift of the calcium transients as the animal approaches and passes the cue under illuminated conditions. These responses are absent in the dark. Thus, widefield cells respond to static stimuli moving across the visual field due to the animal's locomotion. The video was recorded at 10 fps and is played back at 20 fps. Field of view: 1mm x 1mm.
